# Fetal Surveillance in Pregnancies with Myasthenia Gravis

**DOI:** 10.3390/medicina57111277

**Published:** 2021-11-20

**Authors:** Brîndușa Ana Cimpoca-Raptis, Anca Marina Ciobanu, Nicolae Gica, Gheorghe Peltecu, Dan Mitrea, Anca Maria Panaitescu

**Affiliations:** 1 Department of Obstetrics and Gynecology, Carol Davila University of Medicine and Pharmacy, 020021 Bucharest, Romania; brindusa.cimpoca@yahoo.com (B.A.C.-R.); gica.nicolae@umfcd.ro (N.G.); gheorghe.peltecu@umfcd.ro (G.P.); anca.panaitescu@umfcd.ro (A.M.P.); 2 Filantropia Clinical Hospital, 011132 Bucharest, Romania; 3 Neuroaxis, Neurology Clinic, 011302 Bucharest, Romania; dan.mitreal@neuroaxis.com

**Keywords:** myasthenia gravis, pregnancy, arthrogryposis, transient neonatal myasthenia gravis

## Abstract

Myasthenia gravis (MG) is an autoimmune condition, that commonly impacts adult women of reproductive age. Myasthenia gravis in pregnancy is rare, but the incidence is higher in different geographical areas. Pregnancies in mothers with MG can have an unfortunate outcome. Acetylcholine receptor antibodies may pass into the fetal circulation and can affect the fetal neuromuscular junction, generating transient MG or even fetal arthrogryposis. The 2016 and 2021 International Consensus Guidance for Management of Myasthenia Gravis issued by Myasthenia Gravis Foundation of America is lacking in recommendation for fetal surveillance for pregnancies in women with MG. The aim of this paper is to highlight fetal and neonatal complications in mothers with MG and to offer antenatal care insights. Close maternal and pregnancy monitoring can improve pregnancy outcome. Patients with MG should be encouraged to conceive, to avoid triggers for exacerbations of the disease during pregnancy and a multidisciplinary team should be established to ensure the optimal support and therapy.

## 1. Introduction

Myasthenia gravis (MG) is an autoimmune condition, that commonly impacts young adult women (under 40), but it can occur at any age, including childhood. It is not an inherited disease but may be diagnosed in more than one member of the same family. As all autoimmune diseases, MG is characterized by a pathologic response to autoantigens. Its clinical manifestations are the consequence of an error in the transmission of nerve impulses to skeletal muscles due to autoantibodies against nicotinic acetylcholine receptor, or other postsynaptic antigens (muscle-specific tyrosine kinase, low-density lipoprotein receptor- related protein 4, agrin) [1,2] at the level of neuromuscular junction. Usually affected individuals have thymic hyperplasia. Myasthenia symptoms are fluctuating weakness involving variable combinations of muscles: ocular (causing ptosis or diplopia), bulbar (causing impaired speaking, chewing, swallowing), limb, and respiratory [1]. The diagnosis of MG includes detecting the typical antibodies: acetylcholine receptor (AChR-Abs) or muscle-specific tyrosine kinase (MuSK-Abs), however, in a small group of MG patients, these antibodies are absent in the presence of suggestive clinical features (double seronegative MG) while antibodies against low-density lipoprotein receptor-related protein 4, agrin, titin or ryanodine receptors may be demonstrated with suitable assays [2].

**Figure 1:**
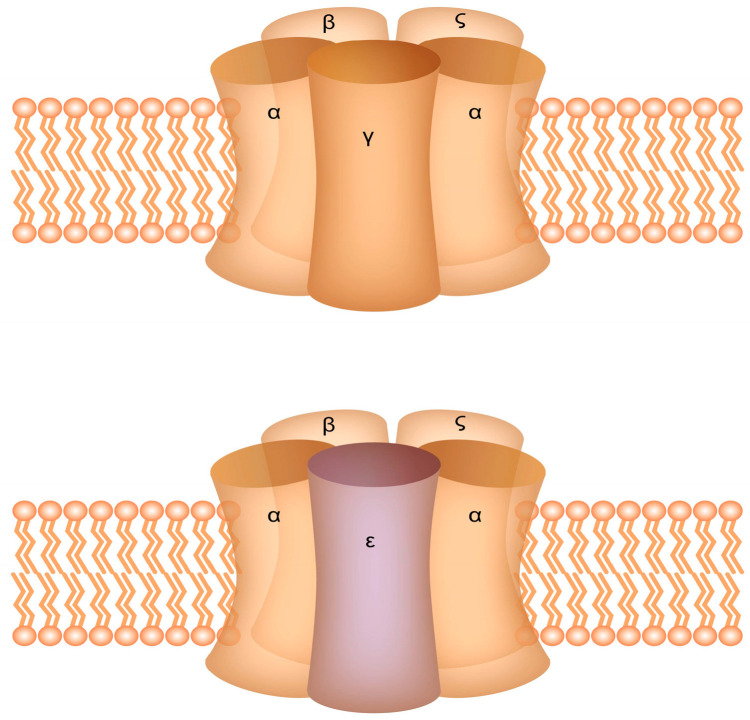
Schematic representation of the Acetylcholine receptor structure—there are structural differences between the fetal-type (**up**) and the adult-type (**down**).

Myasthenia gravis in pregnancy is rare, occurring globally in about 1 in 30,000 pregnant women [3,4], but the incidence is higher in different geographical areas. Pregnancies in mothers with MG can have an unfortunate outcome. AChR-Abs may pass into the fetal circulation by hijacking physiological transfer pathways [5] and can affect the fetal neuromuscular junction, generating self-limited transient neonatal myasthenia gravis (TNMG) (seen in 10%–20% of cases of maternal MG) or, rarely, fetal arthrogryposis multiplex congenita (AMC) (in less than 1% of cases). The AChR in the postsynaptic muscle membrane is found in two isoforms in humans: the fetal-type which is present in the first half of intrauterine life and is replaced by the adult-type which predominates thereafter. The fetal AChR differs from the adult-type in its gamma subunit which is replaced from the third trimester of pregnancy with the epsilon subunit in the adult-type of receptor (Figure 1) [6,7]. It is thought that antibodies responsible for fetal AMC are directed against the fetal-type receptor, while those responsible for TNMG bind to both the fetal and adult types later in pregnancy [8]. For this reason, it may be possible that the diagnosis of a maternal MG would start from the ultrasonographic discovery of a fetus with AMC in an asymptomatic woman. In less severe cases, fetuses exposed to maternal antibodies against the fetal-type receptor, will develop a permanent myopathy known as fetal acetylcholine receptor inactivation syndrome (FARIS) [9].

It is not clear yet whether antibodies associated with seronegative or double seronegative MG are crossing the placenta.

Pregnancies in women with MG are considered high-risk and require intensive monitoring in a multidisciplinary team. The 2016 and 2021 International Consensus Guidance for Management of Myasthenia Gravis issued by Myasthenia Gravis Foundation of America is lacking in recommendation for antenatal care and fetal surveillance for pregnancies in women with MG [10,11].

## 2. Aim

The aim of this paper is to highlight fetal and neonatal complications with MG and to offer antenatal care recommendations.

## 3. Women with Myasthenia Gravis Planning for Pregnancy

Myasthenia gravis is not directly causing infertility, but many affected women of reproductive age postpone or avoid pregnancy. Concerns of possible consequences of their disease or its treatment on pregnancy outcomes, fear of potential worsening of MG due to pregnancy, the uncertainty regarding breastfeeding are the main triggers for anxiety linked to MG and pregnancy [12]. Women with MG should be encouraged to conceive if they wish so. Before pregnancy a consultation with a neurologist and obstetrician (preconceptional counselling) is recommended as treatment options could be changed when planning a pregnancy. Methotrexate and mycophenolate mofetil are contraindicated pre-conceptionally and should be discontinued at least 3 months and 6 weeks, respectively, before conceiving. These drugs should also be avoided in women who might become pregnant or if there is a possibility of pregnancy. Rituximab, a monoclonal antibody that can cross the placenta, recently used in treatment of moderate to sever MG with good results, should also be discontinued at least 6 months before pregnancy as it may cause altered neonatal immune response [13]. Azathioprine and corticosteroids can be continued as they do not seem to interfere with fertility [14]. If a patient is planning for a pregnancy and she did not have a thymectomy, it should be offered; this procedure is thought to optimize disease control and reduce exacerbation of MG [15,16]. Additionally, the risk of TNMG is lower in women who have undergone thymectomy. These protective effects are beginning to show of after at least one year post surgery. Due to its delayed effect, there is no indication for thymectomy during pregnancy [16,17]. In MG thymectomysed patients, vaccines with live-attenuated vaccines (rotavirus, measles, mumps, rubella, varicella, yellow fever) should be carried out only after careful consideration [11,18].

With stable or low activity MG, the risks to pregnancy and the fetus are minimal [1,3]. Most pregnant patients will remain stable throughout gestation and if exacerbations occur, they are more common in the 1st trimester and in the postpartum [19]. The second and third trimester may be marked with a recovery of MG activity, probably due to the physiological pregnancy related immunosuppression [20,21].

Patients should avoid factors that can trigger worsening of the condition that are specific to pregnancy and postpartum, such as infections, aspiration, or medication, especially antibiotics (fluoroquinolones, macrolides), magnesium, and magnesium-containing medications, beta-blockers, and calcium channel blockers [21].

Doctors caring for patients with MG that consider pregnancy or are pregnant are advised to use clinical judgment and the risk to benefit ratio when prescribing drugs that are needed during pregnancy.

Women with MG, as per general population guidelines, are advised to substitute folic acid 3 months prior to conception and during the first trimester of pregnancy. If there is no previous history of neural tube defects, the recommended dose is 400 micrograms [22].

Vaccination before pregnancy is recommended for all women (Table 1). An accurate record of immunizations should be shared with the healthcare provider and when needed, vaccination will be offered before pregnancy. Vaccination with live attenuated vaccines does not affect MG but it is contraindicated in patients on immunosuppressive therapy [10].

Because MG can be associated with other autoimmune conditions, a detailed clinical examination and history review should be carried out, considering testing for antiphospholipid antibodies, thyroid disease antibodies, rheumatoid arthritis, systemic lupus erythematous, or anti-Ro antibodies. These autoimmune conditions can alter the course of pregnancy.

## 4. Surveillance of Pregnancy in Mothers with MG

### 4.1. First Trimester

Prenatal care in pregnant women with MG should be offered in a multidisciplinary team involving a neurologist, psychologist, obstetrician, midwife, fetal medicine specialist, general practitioner, and anesthesiologist. The frequency of the consultations should be adapted to maternal MG clinical status but it must be at least once per trimester.

Routine antenatal booking blood tests should be offered to every pregnant woman with MG and additionally AChR-Abs and MuSK antibodies titers. The levels of AChR antibodies do not correlate with disease severity. Their value is mainly in the initial diagnosis of MG or in the case of modulating antibodies as a potential marker for thymoma in women without thymectomy. The anti-MuSK levels, on the other hand, correlate with the disease severity [25]. If another autoimmune disease is suspected, specific tests are ordered.

Patients with MG should continue the supplementation with folic acid during the first trimester. They should conceive while getting the MG treatment approved in pregnancy. First line treatment are acetylcholinesterase inhibitors (pyridostigmine) for symptomatic relief with dosing adjustment in keeping with the physiological changes occurring in pregnancy: increased renal clearance, increased maternal blood volume and delayed gastric emptying [26]. Corticosteroids are used as second line therapy when adequate doses of pyridostigmine fail to achieve symptomatic relief. For many women these drugs will be maintained throughout pregnancy and postpartum. There is minimal teratogenic risk, a minor raise in the incidence of fetal cleft palate has been reported with the use of steroids in the first trimester [27,28]. Additionally, when there is refractory MG or corticosteroids are not well tolerated, nonsteroidal immunosuppressants, preferably azathioprine or cyclosporine, can be used during pregnancy. In more severe crisis with life-threatening signs, such as respiratory insufficiency or dysphagia, intravenous immunoglobulin (IVIg) or plasma exchange therapy can be considered as a safe, temporary options during pregnancy [11]. Use of eculizumab was recently reported in a pregnant woman with MG [29] with no side effects on the fetus and neonate. Vaccination against Neisseria meningitidis is recommended at least 2 weeks before giving eculizumab, or antibiotic coverage with penicillin [11]. However, until new data become available, use of eculizumab in pregnancy should be limited.

The 11–13 weeks ultrasound scan is useful in assessing fetal anatomy and gives reassurance about normal fetal development, including the appearance of the fetal palate, limbs, joints in mothers with MG on medication. Dating of pregnancy will be made according to the international guidelines: correct measurement of crown-rump length or having the in vitro fertilization details [25]. First trimester screening for chromosomal anomalies, including assessment of the nuchal translucency, should be offered, as per general guidelines. Non-invasive prenatal testing (NIPT) can be offered in pregnancies with MG as the accuracy of the test is not altered by maternal chronic disease, other than neoplasia [30]. We encourage all patients to have a first trimester screening for preeclampsia, including maternal characteristic and first trimester biochemistry and aspirin should be offered to all the high-risk pregnancies [31]. So far, there is no evidence that MG mothers are at higher risk to develop preeclampsia or having a poor first trimester outcome, such as spontaneous abortion or missed miscarriage.

### 4.2. Early Anomaly Scan 16–18 Weeks

An early anomaly scan for patients with MG should be offered, as a reassurance scan. Rarely, maternal MG can cause fetal arthrogryposis multiplex congenita (AMC). This complex condition occurs from the fetal life in <1% of babies of mothers with MG [32]. Fetal AMC is thought to result due to transplacental transfer of maternal autoantibodies against the fetal-type of AChR after 14–16 weeks [5]. The fetal AChR are found in fetal muscles in the early stages of pregnancy and are thought to be replaced by the adult-type by the end of the 3rd trimester. Maternal antibodies against the fetal-type of receptor are causing joint contractures and reduced fetal movements. AMC is characterized by non-progressive contractions in more than two joints and can affect multiple body parts; it may be complicated by severe lung hypoplasia because of abnormal development of the chest and increased amniotic fluid. Most AMC cases diagnosed in the fetal life occur due to genetic and chromosomal abnormalities, brain and spinal abnormalities, oligohydramnios, or viral infections, and are not linked to maternal MG [33], however, sometimes, the diagnosis of MG in a asymptomatic woman can start from the ultrasound findings of fetal AMC [34].

Prenatal ultrasound diagnosis can be made as early as 16–18 weeks. Despite stimulation, the fetus does not show movements on ultrasound examination and the joints are in an abnormal position, either fixed flexion or fixed extension. The disease is more severe when multiple joints are involved. For those infants that are born with AMC the prognosis is unfortunate as the condition is persistent and progressive. The associated lung hypoplasia can lead to respiratory failure and death. An early ultrasound identification of the condition gives parents reproductive choices, including termination of pregnancy.

### 4.3. Routine Anomaly Scan 20–24 Weeks

**Figure 2:**
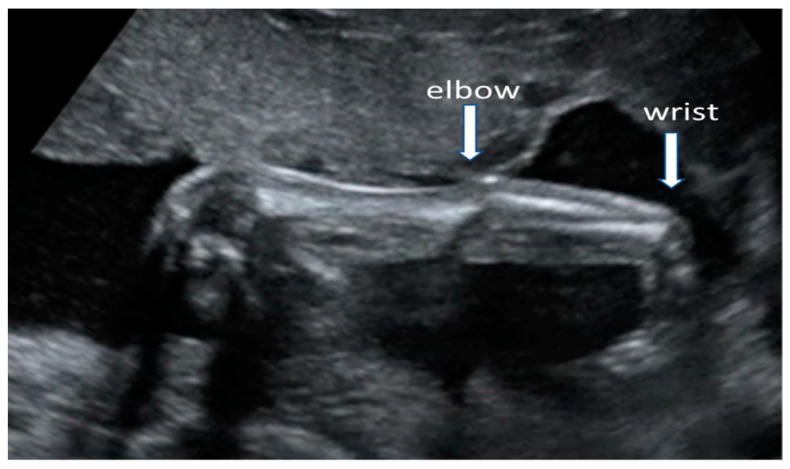
Ultrasound scan of a 22 weeks fetus with arthrogryposis multiplex congenita showing extended right upper extremity with fixed joints: elbow in extensions and wrist in flexion.

The routine anomaly scan should be offered in all women with MG as most major fetal abnormalities can be diagnosed at this gestational age or chromosomal abnormalities can be suspected based on ultrasound findings [35]. Women with MG do not have a higher risk of having a baby with genetic disorders or structural defects, other than AMC (Figure 2). Fetal wellbeing will be defined after assessment of growth, anatomy, fetal movements, and amniotic fluid volume.

If a chromosomal or genetic condition is suspected after the ultrasound examination, diagnostic invasive procedures should be offered to women with MG: during the first trimester chorionic villus sampling and during second trimester amniocentesis [36]. The risk of miscarriage is the same as in any other pregnant woman. All pregnant women with MG, like any other pregnant woman, should be offered a mid-pregnancy assessment of the cervix, as part of the screening strategy for preterm birth.

### 4.4. Third Trimester Serial Growth Scans

Pregnant women carrying the antibodies against the fetal AChR are often asymptomatic for MG as these types of antibodies do not attach to the adult for of the AChR that is usually found in maternal muscles [37].

Every pregnant woman with symptomatic or asymptomatic MG who attends the hospital with reduced fetal movements or complaining of rapid raise of abdomen size should have an ultrasound examination and the appearance of fetal joints should be assessed together with amniotic fluid volume. If polyhydramnios in a pregnancy within a baby with fixed joints is diagnosed, the suspicion of AMC is made.

Depending on the clinical circumstances an ultrasound scan every 2–4 weeks in mothers with MG, to assess fetal growth, wellbeing, movements and amniotic fluid volume could be offered. The routine antenatal vaccination scheme should be followed also for patients with MG (Table 1).

### 4.5. Intrapartum Care

Pregnancy risk must be stratified based on maternal and/or fetal complications and not by MG alone. Intrapartum monitoring is recommended for all pregnancies with mothers affected by MG. Vaginal delivery is the method of choice; caesarean section (CS) should be carried out only if there are obstetric indications. Locoregional anesthesia is preferred, mainly epidural block. It is advised to optimize pain control and to avoid unnecessary maneuvers as exertion and stress may trigger myasthenic crisis [38].

Instrumental vaginal delivery is more common in mothers with MG to prevent prolongation of pushing and maternal fatigue. The uterine muscles are smooth and, therefore, are not affected by MG and their contractility is maintained during labor. Skeletal maternal muscles, that might be weakened because of MG, contribute to the second stage of labor which require maternal pushing effort. If pushing is not effective, assisted delivery may be required [39].

### 4.6. Postpartum Care

During pregnancy, the clinical course of MG is difficult to predict, but as any other autoimmune disorder, it can worsen after birth [19]. Postnatal consultation with the multidisciplinary team is essential as treatment doses might need to be change.

Breastfeeding should be avoided for newborns with TNMG since AChR-Abs can be transferred to the baby through milk. Anticholinesterase inhibitors (pyridostigmine and neostigmine) do not pass into breast milk, therefore this treatment is safe in breastfeeding. It is recommended that mothers under azathioprine or mycophenolate avoid breastfeeding [40].

### 4.7. Neonatal Surveillance

Mothers with MG have a risk of 10%–20% of having a child affected by TNMG. This risk is higher in women with exacerbations of myasthenic symptoms and in those reluctant to treatment, or those without thymectomy. About 70% of affected babies will develop symptoms within the first day of life and rarely up to 4–7 days [41]. During the first 3 weeks after birth the maternal antibodies are cleared out of the neonatal circulation [42]. Neonatal MG symptoms are usually mild or moderate: poor sucking, palpebral ptosis, facial paresis and/or generalized hypotonia. Respiratory support and tube feeding are only rarely necessary. Close monitoring of the neonate is vital to detect involvement of respiratory or swallowing muscles. This condition is transient, and it spontaneously remits, however, is some cases, high-risk nursery could be considered.

## 5. Discussion

From the maternal-fetal specialist perspective, MG is a condition compatible with pregnancy and women affected by this autoimmune disorder should be encouraged to conceive if they are contemplating a pregnancy. Planning for pregnancy when the disease is adequately controlled and multidisciplinary approach involving an obstetrician, neurologist, anesthesiologist, neonatologist are the two main factors that contribute to a favorable outcome. Additionally, these pregnancies should be ideally followed-up in specialized centers where physicians experienced in treating MG are available. Most women remain stable during pregnancy if their symptoms are minimized before conception and worsening occurs rarely after delivery. Outside pregnancy there are many effective treatment options available and many of them can be continued during pregnancy and breastfeeding, with few exceptions referring especially to mycophenolate mofetil and methotrexate (Table 2). Antepartum care for women with MG is mostly the same as for healthy patients unless fetal complications are detected, and the maternal condition is stable; closer surveillance is indicated. The severe form of fetal complication, arthrogryposis, is thought to result from transplacental passage of antibodies against the fetal AChR; women carrying the fetal-type antibodies are often asymptomatic, and the risk for subsequent AMC affected pregnancy may be as high as 100% therefore counselling is very important. Delivery is decided based on obstetrical criteria, aiming for vaginal birth. Postnatal evaluation is necessary for all neonates in order to exclude any transient myasthenic symptoms, mainly feeding and respiratory difficulties, even if the patient’s condition was well controlled during pregnancy. Additionally, the mother requires close monitoring after birth due to a higher risk of symptoms exacerbation during the next weeks after delivery.

## 6. Conclusions

Maternal and pregnancy close monitoring in a multidisciplinary team can improve pregnancy outcome for women with MG. Patients with MG should be encouraged to conceive, to avoid triggers for exacerbations of MG and a multidisciplinary team should be established to ensure the optimal support and therapy. The risk for fetal arthrogryposis is less than 1% and for TNMG of 10%–20%. The course of MG is sometimes unpredictable, but many pregnant women remain stable.

## Figures and Tables

**Table 1:** Vaccines in women with MG recommended during pregnancy and breastfeeding.

Vaccine	Recommendations
Whooping cough (Pertussis)	27th–36th week of pregnancy [23]
Influenza (September–March)	—inactivated influenza vaccine- intramuscular injection [24]
COVID-19 vaccination	—at any time in pregnancy or after, ideally with a m-RNA vaccine
Vaccines for travel	4–6 weeks before trip, to be reviewed by healthcare providers [23]

**Table 2:** Treatment recommendations during pregnancy and breastfeeding.

Treatment	Recommendation during Pregnancy/Lactation	Recommendation during Lactation
Pyridostigmine	First line treatment, safe Intravenous anti-cholinesterase inhibitors should be avoided during pregnancy because they can induce uterine contractions. Can be used intravenous during labor	Safe in breastfeeding
Corticosteroids	First option as immunosuppressive, slightly higher risk of cleft palate; gestational diabetes, prematurity	Safe during breastfeeding
IV immunoglobulin	Safe, used in myasthenic crisis	Safe, used in myasthenic crisis
Plasma exchange	Safe, used in myasthenic crisis	Safe, used in myasthenic crisis
Azathioprine	Continuation can be considered	Can be taken into consideration
Cyclosporine	Can be associated with prematurity, low gestational birth weight	Generally considered safe
Tacrolimus	Conflicting evidence, generally regarded safe	To be avoided
Mycophenolate mofetil, Methotrexate, cyclophosphamide	Contraindicated, discontinuation before conception	Contraindicated
Rituximab	Limited data, generally not advised, associated with neonatal immune alteration	Limited data, not used
Eculizumab	Limited data	Limited data

## Data Availability

Not applicable.
